# An Account of the Dysentery, as It Appeared among His Majesty's Troops in Jamaica during the Late War; with Dissections Explaining the Proximate Cause of That Disease; and a More Simple and Efficacious Method of Treatment Thence Resulting Described

**Published:** 1786

**Authors:** Thomas Cawley

**Affiliations:** late Surgeon to His Majesty's Military Hospital in Jamaica


### LONDON MEDICAL JOURNAL,

FOR THE YEAR 1786,

### PART THE FOURTH.

I. *An Account of the Dysentery, as it appeared among His Majesty's Troops in Jamaica during the late War; with Dissections explaining the proximate Cause of that Disease; and a more simple and efficacious Method of Treatment thence resulting described.— Communicated in a Letter from Mr.* Thomas Cawley, *late Surgeon to His Majesty's Military Hospital in Jamaica, to* Robert Adair, *Esq. Surgeon General to the Army, and by him to Dr.* Simmons.

*To* ROBERT ADAIR, *Esq.*

SIR,

HEREWITH I send you an account of the dysentery, as it appeared among His Majesty's troops in Jamaica during the late war. If you think it contains any information on the subject worthy of being laid before the public, I beg the favour of you to communicate it to Dr. Simmons, to be inserted, if he thinks proper, in the London Medical Journal; in which case I shall be encouraged to send you a similar account of the yellow fever of the West Indies, a disease concerning which authors have hitherto been very imperfect or mistaken.

I am, Sir,

Your much obliged,

Humble Servant,

THOMAS CAWLEY.

*Chester*,

Sept. 20, 1786.

The subjects of the following cases belonged to the 85th, 92d, 93d, and 94th regiments, which were landed at Kingston, in Jamaica, in August, 1780, after having been on board transports, on their voyage from England, about five months. During this long and crowded confinement, incapable of practising those means which experience has found useful in preventing diseases at sea, and likewise in a great measure unprovided with the necessaries thereto concurring, several were attacked with fevers, and, on landing, very few were free from scurvy, and many of them were in a very deplorable state of that destructive disease.

A general

A general hofpital was fitted up at Kingfton for the reception of fuch of the fick as could not be accommodated in regimental hofpitals: it was attended by Mr. Cudlipp and myfelf; and this inftitution gave me the opportunity of making the following, and many other diffections. They are probably more than have ever fallen to the lot of any one practitioner, the annexed being only about a fourth part of the cafes of dyfentery in which the morbid parts were examined after death; the appearances having been invariably the fame, a greater number was deemed unneceffary to elucidate the pathology of the difeafe.

From Auguft to January, of two hundred and forty-nine patients who were admitted under my care, one hundred and feventeen had fevers, and ninety-three fluxes; of the former, thirty-one died; of the latter, fifty-one; or more than one half: the fatality was in the fame proportion throughout the hofpital, where about two hundred fluxes were admitted within the fame period. It muft be remarked of thefe cafes, that they were generally of feveral days or weeks ftanding, and confequently the difeafe was become deeply rooted: the patients were likewife debilitated by fevers, and in almoſt every inſtance were ſcorbutic; both which ſtates ſeem to be ſo powerful in prediſpoſing to flux, that in thoſe admitted under theſe circumſtances only, the diſeaſe was almoſt ſure to ariſe, though placed in remote parts of the hoſpital, where they had no communication with the infectious; whilſt the healthy, though immediately expoſed, by lying near to, or attending, the ſick, eſcaped. Some inſtances occurred of a diarrhœa, which ended in dyſentery, being brought on by a doſe of jalap, where the patient was conſiderably ſcorbutic and debilitated; and I have obſerved the ſame effects from a doſe of ſalts given to a ſcorbutic patient. When the ſcurvy, contracted at ſea, diſappeared in the army, the dyſentery likewiſe abated in frequency and fatality.

In March, 1781, I found fifteen patients ill of the chronic dyſentery in the regimental hoſpital of the 92d regiment, at Spaniſh Town, of whom four died. From March to January only eleven patients with fluxes were admitted into the ſame hoſpital on the firſt attack, and all recovered, though within the eight preceding months one half of that regiment had fallen a ſacrifice to dyſenteries and fevers. In what proportion theſe two diſeaſes committed

ſuch

ſuch ravages was not aſcertained, but it may be preſumed to have been the ſame as happened in the general hoſpital. The mortality was not leſs in any of the three remaining regiments. In the 93d it was much greater, and in all from the ſame cauſes. It is a fact that upwards of half of the men belonging to thoſe four regiments died within eight months after their landing; and as fevers and fluxes conſtituted eight tenths of the diſeaſes which prevailed within that ſpace of time, and as fevers in the general hoſpital were only fatal to a little more than one fourth, whilſt the dyſentery was fatal to more than one half, the number attacked with each being nearly the ſame, we may conclude, without any great riſk of error, that the number of deaths from dyſentery alone exceeded the number from every other cauſe within the eight months above mentioned.

As a farther proof that this prevalence and fatality of the dyſentery were cauſed by the ſcurvy, we ſhall juſt mention the ſucceeding ſtate of the regimental hoſpital of the 92d regiment. Three hundred and thirty-one patients were admitted between March 1ſt and December 31ſt, 1781. Of theſe, one hundred and fifty-four had fevers, and eleven had fluxes, but but not one was fcorbutic. The remainder, excepting twelve, had fmall-pox by inoculation, dry belly-ach, and ulcers. The fame change took place about the fame time in all the four regiments.

The fcurvy and its effects would have ceafed fooner, if the foldiers, on landing, had been immediately put on a full allowance of frefh provifions, in lieu of rations, to which they were in a great meafure confined, with the fcanty addition of vegetables which the furplus of their pay enabled them to procure.

From the beft information I could get, the fcurvy did not appear in any confiderable degree until about fix weeks before their landing; and it is probable, from the following fact, that it never appears in lefs time than ten weeks after expofure to thofe caufes which are moft powerful in producing it.—On the 10th of November, 1783, I failed from Port Royal on board a merchant fhip hired to carry home invalids, &c., wherein were embarked about three hundred and twenty perfons, of all defcriptions. Between decks they were prodigioufly crowded, above fifty more than were contracted or provided for having been fent on board. On the 17th of January we arrived off Dover, and within within the next ſeven days all the men were diſembarked in a very good ſtate of health, not more than five or ſix being ſcorbutic, and thoſe very ſlightly. Their diet conſiſted, as uſual, of ſalt proviſions, a few only being provided with yams, cocoas, and fruit. The vinegar, laid in by Government, was expended before the voyage was half completed; and as the tranſition from great heat to extreme cold was too ſudden to be borne by men relaxed by long-continued heat, and very lightly clothed, but few were to be ſeen on deck within the laſt three weeks of the paſſage, which occaſioned a prodigious accumulation of filth, and a horrid ſtench.

From this brief account of the ſcurvy, and its conſequences, we ſhall juſt remark, that, independent of motives ariſing from humanity, if we conſider the ſubject only in a political view, the prevention of ſcurvy ſeems to be an object of great importance; for no one ſurely will argue, that the expence of preſerving health, in an army or fleet abroad, can be put in competition with the national ſecurity and intereſt depending on their operations, excluſive of the loſs of time, and expence of ſupplying every individual loſs.

It

It is commonly objected to diſſections, that they demonſtrate effects rather than cauſes; but if we conſider that the proximate cauſe of dyſentery is ſecondary, or produced by an inviſible, unintelligible, remote cauſe, which by none of its qualities is ſubject to our ſenſes; and farther, that our chief aim in curing this diſeaſe conſiſts in an attempt to obviate the effects, it follows, that the farther knowledge we acquire by diſſection of ſuch effects in every ſtage of the complaint, the nearer we are likely to arrive at a rational and ſucceſsful method of cure.

### CASE I.

J. B. aged twenty-three years, was admitted, Auguſt 23d, with a diarrhœa, of a fortnight's ſtanding, which, two days after his admiſſion, turned to flux. This continued, without any fever or great violence, till September 23d, when he died, greatly emaciated.

The treatment conſiſted of warm, opiate, aſtringent, and cordial medicines, with now and then an opening draught of ſalts and oil; by which means the evacuations were every now and then checked, but ſoon returned with increaſed griping and frequency.

On diffection the ileum was found confiderably inflamed at its inferior extremity; the right extremity and arch of the colon were quite found, but the left extremity was externally much inflamed, contracted, very hard, and felt as if fchirrhous, and in two places was perforated by ulceration.—That part, where the colon and rectum join, was found; but the rectum was externally inflamed, very hard, and within in a gangrenous ftate.

### CASE II.

W. Evans, aged forty-five years, admitted Auguft 20th, had had a fever two months before, and now complained of fcurvy in his mouth, with fwelling of his legs, which were hard and blue.—Auguft 27th a purging came on, which turned to dyfentery, under which he lingered, with now and then a flattering profpect of amendment, until November 17th, when he died, greatly emaciated.— Every fecond or third day, at the beginning, he took falts and oil, and on the intermediate days vitrum antimonii ceratum: afterwards opiate, diaphoretic, and aftringent medicines, with ftarch clyfters; and laftly, rhubarb and ipecacuanha to procure natural ftools, and the bark to corroborate.

After death the ileum, at its inferior extremity, was found much inflamed; the arch of the colon, on the left ſide, and the rectum externally, were a little inflamed, contracted, hardened, and internally ulcerated: between the colon and rectum the gut was burſt.

### CASE III.

J. Brown, aged twenty-five years, was admitted, Auguſt 21ſt, with a diarrhœa.—On the 23d he was ſeized with fever, and had an increaſe of the purging.—On the 29th he was free from complaints; but within a few days a dyſentery came on, and, without any return of fever, he died September 24th.

In this caſe ſalts and oil were given occaſionally; white decoction for common drink; every night a draught, with ſp. mindereri and tinct. thebaic; and when not under the operation of evacuants, the patient took a mixture of infuſ. corticis Peruv. Confect. Damocratis and tinct. Japonic. with ſtarch clyſters.

On diſſection the ſmall inteſtines were found to be extremely vaſcular, appearing as if injected; the left extremity of the colon, and rectum externally, was inflamed, contracted, and hard; the villous coat every where ulcerated, rated, and in places gangrened.—The diseased part of the colon formed an uncommon *lusus naturæ*; at the great extremity of the stomach it mounted upwards and backwards, forming an angle, whose convex part was strongly attached to the diaphragm between the spleen and vertebræ; it was also strongly attached to all the parts in contact with it by inflammatory exudation.

### CASE IV.

Thomas Havers, aged twenty-six years, admitted August 31st, had been labouring under a flux three weeks, was scorbutic, and had swelled legs.—The dysenteric symptoms left him two or three times for a day or two, but returned with increased violence. October 2d his left leg and thigh were very much swelled, and œdematous, and on the 7th he died.

At first salts and oil were administered, with decoct. alb.—pulv. Dovari ʒj. h. s., and starch clysters; lastly, infus. amar. c. scillis, and pills of soap and rhubarb.

The abdomen contained two pints of water; the left side of the colon and rectum, externally, was not inflamed, but they were contracted to a very small diameter, felt very fleſhy, and in places knotty; internally they were ulcerated conſiderably, and beſmeared with blood mixed with pus. The meſentery was remarkably thick and fleſhy, and appeared as if injected. Near the bifurcation of the aorta was a large cluſter of indurated lymphatics, and a chain of them accompanied the left arteria iliaca externa as far as the groin.

### CASE V.

Thomas Cookes, aged fifty-four years, admitted Auguſt 31ſt, had had a purging five weeks, and at the time of his admiſſion went to ſtool twenty times a day. September 6th it ſtopped, but returned on the 13th. On the 25th his ſtools became white, without gripes. October 1ſt, 2d, 3d, he had yellow ſtools, without gripes. October 24th the purging was profuſe, but without gripes, and the ſtools reſembled pus. November 1ſt he was better of the purging, but his belly began to ſwell, and he died on the 20th, the purging having nearly left him ſeveral days before his death.

The treatment in this caſe conſiſted at firſt of cathartics, with a diaphoretic opiate draught at night; afterwards, of bitter, aſtringent, and cordial medicines, with pills of ſoap and ſquills, and pulv. Dovari ʒj. h. f.; and, for common drink, a decoction of ftarch with g. arabic.

On diffection the omentum was found to be nearly confumed. The abdomen contained about two gallons of water. The colon, on the left fide, was internally flightly ulcerated in feveral places, apparently the remains of large ulcers. The fpleen was full of fchirrhous tumours, and its convex furface covered with a white fcale, which, in the center, was of a bony confiftence.

### CASE VI.

Mrs. Northop, one of the nurfes, was feized with dyfentery September 10th, and died on the 17th. The griping was violent, and the dif-charge very frequent and bloody, with conftant tenefmus.

The colon, from one extremity to the other, was externally a little inflamed; its coats thic-kened; the whole internal coat, from cœcum to rectum, gangrened, and highly putrid: the inferior extremity of the rectum was inflamed.

### CASE VII.

Thomas Walker, admitted Auguft 29, aged twenty-eight years, had a fever at fea, and then became became fcorbutic. His gums were nearly confumed; his knees and ancles fwelled, hard, and blue. He was put on a courfe of vegetables, with bark and elixir of vitriol. September 10th he was feized with dyfentery. On the 16th he was feverely griped, had tenefmus and bloody ftools, and died that evening.—The omentum was found inflamed, and beginning to be fchirrhous; the duodenum, jejunum, and ileum, at their attachment to the mefentery, were a little inflamed. The inflammation increafed downwards, fo that at the inferior extremity of the ileum its internal coat was beginning to gangrene. The right extremity of the colon was externally inflamed, and internally gangrened. In the middle of that gut there was lefs difeafe, and on the left fide none. The rectum was in the fame ftate as the right extremity of the colon.

### CASE VIII.

Matthew Neil, aged feventeen years, admitted Auguft 31ft, had had a fever at fea, and had laboured under a flux ten days. His ftools were flimy and bloody, with tenefmus and *procidentia ani.* The violence of thefe fymptoms in a few days abated, and the ftools not only became became lefs frequent and lefs painful, but white. September 29th he died, greatly emaciated.

In this cafe, at firft, on alternate days, an oily cathartic was given; on the intermediate days fmall dofes of ipecacuanha, and an opiate at night. Afterwards, the infuf. cort. Peruv. c. confect. Damocrat. and tinct. Japonic. with a little rhubarb now and then to procure natural ftools, were adminiftered, with the decoct. amyli and g. arabic for common drink; and ftarch clyfters.

On diffection the rectum and left extremity of the colon, as high as the arch, appeared, externally, a little inflamed: their diameter was contracted, their coats were confiderably thickened, and they felt flefhy, but were free from indurations; their internal coat was all the way corroded and ulcerated: the whole furface prefenting flefhy, fungous excrefcences, which appeared to be only the natural rugæ ftripped of their villous covering, and partly confumed in places.

### CASE IX.

Richard Davis, aged thirty-one years, admitted Auguft 29th, had had the dyfentery three months before this period, and ftill remained fcorbutic, his legs being hard and blue. September 7th he was attaeked with gripes and bloody ftools. He took falts and oil, or rhubarb and vit. antim. cerat.—an opiate h. f.—the decoct. arabic. for common drink, and had ftarch clyfters injected.

October 1ft, the purging having left him, bitters and aftringents were adminiftered.

October 7th, having had feveral bloody ftools in the night, he took vit. antim. cerat. and rhubarb again.

October 15th he was free from purging, and the bitters and aftringents were repeated. The frequency of ftools, however, returned, and he continued to have fix or eight (confifting of mucus and pus) every day until the 22d, when he died, exceffively emaciated.

The ileum was found much contracted and inflamed; the left extremity of the colon was contracted, and externally interfperfed with livid fpots, but not mortified; and it felt hard and flefhy. The internal coat was ulcerated, and interfperfed with tumours of a blue caft. The rectum was in a worfe ftate, inclining to gangrene. In the jejunum were two intuffufceptions, without inflammation. The gall bladder bladder was confiderably diftended, and adhered to the epiploon.

### CASE X.

Samuel Howel, aged twenty-feven years, was admitted October 1ft for a relapfe into dyfentery. His ftools, at firft, were flimy, with gripes. On the 20th he was feized with fever, gripes, and bloody ftools, and died on the 23d.

The ileum was partially a little inflamed; the colon, on the left fide, was contracted, hard, and in places inclining to lividity, which refembled that of the legs in fcorbutic patients; the villous coat was ulcerated. The rectum was in a worfe ftate, its villous coat being gangrened. The abdomen contained a little yellow water. The vefica fellea contained a ftone about the fize of a nutmeg, and was thickened where the ftone lay; it likewife adhered to the epiploon and right extremity of the ftomach.

### CASE XI.

Thomas Pepper, aged twenty-four years, admitted October 27th, had had a fever and fcurvy, which were fucceeded by purging, of two months ftanding when admitted. His ftools were frequent, and white, with gripes. These symptoms increasing, he died on the 30th.

The abdomen contained about a gallon of water. The left extremity of the colon and rectum was of its natural colour, but contracted and hard. The internal coat was ulcerated. The mesentery was thickened, and of a fleshy appearance. The glands, about the receptaculum chyli, were very numerous, enlarged, and inflamed.

### CASE XII.

Aaron Akeroyd, aged twenty-six years, was admitted August 30th. September 1st a purging came on, which became white soon afterwards, then brown, and yellow, but much too frequent, and accompanied with gripes. At the latter end of October these symptoms were much increased. On the 6th of November the stools became bloody, and very frequent, and he died on the 17th.

The colon was contracted to a very small diameter; internally were only the marks of past disease, the ulcerations being healed. The flux had entirely left him six days before his death, during which time he lay almost senseless and speechless.

### CASE XIII.

Henry Craig, aged twenty-three years, admitted October 28th, laboured under a flux thirteen weeks. His ſtools were ſometimes bloody, ſometimes white. November 1ſt they became involuntary, and he was always ſleepy. On the 12th he died, greatly emaciated.

The left extremity of the colon was contracted and hard. There was no appearance of external inflammation. The internal coat was ulcerated, and in places oozed out blood in conſiderable quantity. The rectum was in a ſtill worſe ſtate.

### CASE XIV.

Joſeph Mellows, aged twenty-one years, admitted October 25th, was ſcorbutic, and labouring under a flux, which had continued ſix weeks. He had ſeveral white ſlimy ſtools every day. He died on the 12th of December.

The colon and rectum were contracted and hard, without any external inflammation, but their internal coat was ulcerated.

### *Extracts from Authors relative to the Cause and Seat of Dysentery.*

Sydenham, after having attentively consider-ed the various symptoms attending this disease, "discovered it to be a fever of its own kind, "turned inwards upon the intestines, by means "of which the hot and sharp humours that "were contained in and agitated the blood, "were thrown off by the meseraic arteries upon "these parts, whence blood was discharged by "stool, the mouths of the vessels being open-"ed by the impulse of the blood and humours "flowing thereto *."

Morgagni relates †, that Valsalva, in a case of dysentery, found a great quantity of sanious ichor in the belly, which issued out of the in-testines in many places, where they were per-forated to some considerable extent. This part comprehended the extremity of the ileum, and the adjoining part of the colon, to the ex-tent of two hands breadth.

In another case ‡, (a patient of Platerus) quoted by the same writer, innumerable little

* Swan's Translation of Sydenham's Works, page 157.

† De Sed. et Cauf. Morb. Epist. 31, art. 2.

‡ Ibid. art. 15.

ulcers were found on the whole extent of the surface of the ileum, three fingers breadth diſtant from each other; and Morgagni adds, that Baſſius relates a ſimilar hiſtory. Nothing is ſaid of the ſtate of the colon in theſe caſes, which might have been overlooked for want of external inflammation.

The ſame author obſerves, that bodies conſiſting of fat in appearance, and bodies ſeemingly fleſhy and membranous, may be equally diſcharged from the inteſtines without any ulcer affecting them, they being of the nature of polypi, and formed by the blood ſtagnating in the cells of the colon, where it concretes, and the red parts ſubſide. Sometimes, he adds, the internal membrane ſloughs off.

Theſe bodies, he farther obſerves, which are diſcharged by dyſenteric patients in form of membranes, ſometimes are real membranes, but often falſe, and no proof of ulceration. They may ariſe, he thinks, from anaſtomoſis, owing to an increaſed impetus and relaxation of the extremities of the arteries. He is of opinion that teneſmus is cauſed more frequently by the acrimony of the mucus and blood ſtagnating in the cells of the colon than by inflammation of the the rectum: and he obſerves *, that inflammations of the inteſtines have often terminated in gangrene, without any conſiderable pain or fever.

Sir John Pringle †, on opening a man who died of dyſentery three weeks after the attack, found the larger inteſtines black and putrid, the coats preternaturally thick, and internally much ulcerated, eſpecially the rectum and lower part of the colon. The villous coat was either abraded, or changed into a corrupted ſlimy ſubſtance, of a greeniſh colour, not only in the part deſcribed, but alſo in the coecum and its appendix. In another patient ‡, who died on the 7th day, he found the rectum extremely putrid, and from thence the gangrene ſeemed to have ſpread to the colon, which was entirely mortified, but chiefly towards its lower end. The villous coat was partly conſumed, and what remained was blackiſh, tender, and eaſily ſeparated. The vaſcular coat had the appearance of a preparation well injected. The ligaments which contract the colon longitudi-

* Epiſt. 35.

† Obſerv. on Diſeaſes of the Army, 6th edit. p. 238.

‡ Ibid. p. 239.

nally; and form the cells, were half corrupted, and adhered loosely to the outer coat; part of the cœcum was also mortified, but the rest, with the small intestines, was of a firmer texture, and only inflamed.

Dr. Monro, in his work on the Diseases of Military Hospitals, says, that, in those who died of dysentery, the rectum was always found inflamed, and partly gangrened, especially its internal coat.—In two subjects the lower part of the colon was inflamed, and there were several livid spots on its great arcade. In one whose body was much emaciated, and who was seized with a violent pain of the bowels two days before death, all the small guts were red and inflamed, and in another there were livid gangrened spots on the stomach.

Dr. Cullen, speaking of the proximate cause of dysentery*, supposes that the whole, or at least the chief part of it, "consists in a preter-"natural constriction of the colon, occasion-"ing at the same time those spasmodic efforts "which are felt in severe gripings, and which "efforts, propagated downwards to the rectum,

* First Lines of the Practice of Physic. § 1078. 4th edit.

"occasion

" occasion there the frequent mucous stools

" and tenesmus."

The pathology of dysentery deducible from the foregoing extracts is contradictory and undetermined; and as the method of cure has consequently been fluctuating, it is hoped that any progress made towards setting the subject in a clearer light will have a tendency still to improve the treatment.

From the dissections it is evident that the internal or villous coat of the rectum and colon is the true seat of dysentery, and that inflammation and its consequences there excited, essentially constitute the whole of it. The rectum being generally found in a worse state than the colon, and the extremity of the colon adjoining to the rectum being the only part of that gut which in general partakes of the disease, it may be inferred that the disease begins in the rectum, and spreads to the colon in the greater number of instances, and that inflammations in other parts of the alimentary canal are only accidental complications.

If the inflammation be moderate, and no pyrexia attend, it consists of partial phlegmasiæ, or efflorefcences, which terminate in ulcers in a few days; this was by far the moft common termination; but when the inflammation is violent and extenfive, accompanied with pyrexia, a mortification and fudden death enfue. The dyfenteric inflammation of the villous coat of the rectum and colon, though arifing from a fpecific caufe, terminates therefore like all other inflammations — a falutary one by refolution, a fatal one by gangrene, and one of very doubtful event by fuppuration and ulceration. It is not uncommon to find all thefe appearances in the fame cadaver. *e. g.* the villous coat of the rectum gangrened, higher up ulcerations, and above that, in many different parts of the colon, fmall efflorefcences proceeding towards ulceration. — How far does this defcription agree with what Dr. Cullen terms erythema, Vol. I. p. 154 of his Firft Lines, and in his Synopfis Nofologiæ, G. vii. Sp. 2.?

The next changes the inteftines undergo are a very confiderable contraction of their diameter, and thickening of their coats; the firft perhaps from pain inducing fpafm, the fecond from an increafed derivation to the parts affected in confequence of pain.

 The

The nature of the remote caufe of dyfentery is very obfcure, but it feems in all cafes and climates to be the fame fpecific poifon; difference of temperament, climate, feafon, fituation, &c. making only a variety in the progrefs of its effects, but by no means changing its effence.—If the remote caufe or virus which occafions dyfentery were generated in the blood, it would be extremely difficult to conceive how it can be invariably determined to the villous coat of the rectum and colon. Were the determination to the bowels general, the difeafe might more readily be fuppofed to be *vicarious*, or, as Sydenham called it, *Febris introverfa*; but how the morbific matter comes to be difcharged through the mefenterica inferior only, and depofited on the villous coat of the rectum and colon, is a problem which I cannot pretend to folve. Is it not more probable that it is taken in by deglutition, or otherwife produced in the alimentary canal*?

The only evident predifpofing caufes were, fcurvy, and debility from fevers; thofe who were

* This difeafe, like many others, has been attributed to the united influence of a variety of caufes, as if one were infufficient, which has rather contributed to fhow our own ignorance than were in thefe ftates were not only very apt to catch the difeafe, if expofed to infection, but in fuch it appeared very often fpontaneoufly to arife where no infection could well be fufpected, which tends to fhow that the fpecific contagion may be occafionally generated.

Dr. Cullen* defines dyfentery thus: — "Pyrexia contagiofa; dejectiones frequentes, mucofæ, vel fanguinolentæ, retentis plerumque fœcibus alvinis; tormina; tenefmus."—As to the pyrexia, it muft be obferved that in 1781, 1782, and 1783, the dyfentery was by far moft frequent in autumn, after the heavy rains of that feafon; it fometimes mixed with the bilious, intermitting, and remitting fever of that feafon, fometimes alternated with it, but much more frequently was a confequence than concomitant. It frequently exifted long before any febrile fymptoms occurred; often thofe fymptoms never fupervened; and in cafes accompanied with pyrexia, if not fuddenly fatal by fupervening gangrene, the dyfentery continued than to throw light on the fubject. Is it not much better to fay at once, that the caufe is *unique*, or fpecific, and incomprehenfible?—Why not, as well as in the fmall-pox, meafles, &c.?

* Synopf. Nofol. Meth. Cl. I. O. v. G. xli.

 long long after the fever had fubfided. From thefe confiderations *, I apprehend that fever is not effential to dyfentery, but a cafual occurrence from independent caufes, or in fome cafes fympathic of its violence. Moreover, it was never epidemic among the troops, *i. e.* never originated from any general caufe exifting in the atmofphere. Its contagious power was likewife very limited; no inftance occurred of a furgeon catching it, and only one of a nurfe †; whether fhe was predifpofed by fcurvy or debility was not noticed. — Zimmerman, fpeaking of the epidemic dyfentery in Switzerland, in 1766, obferves, that it was not of itfelf contagious. The contagious power, he fays, lay chiefly in the excrements; fo that, according to him, it becomes fo chiefly from naftinefs, and crowding too many people together; and Sir John Pringle obferved, that the infection was evidently communicated by the fæces of thofe

* "It fometimes begins with a chilnefs and fhaking, im-
"mediately fucceeded by a heat of the whole body, as is
"ufual in fevers, and foon after gripes and ftools follow: it
"is indeed frequently not preceded by a fever, but the gripes
"attack firft, and ftools foon fucceed."

*Swan's Tranflation of Sydenham's Works, p.* 152.

† Cafe VI.

ill ill of the diftemper. Hence we may conclude, that the contagion is not fo extenfive in its influence as has been imagined, where a predifpofition does not exift.

*Prognofis.* — If the patient be healthy at the time of attack, and a proper treatment entered on without delay, the danger is not confiderable; but if dyfentery fucceeds fever in a debilitated habit, or if the patient be old or fcorbutic, and the difeafe of long ftanding, the probability of recovery is very remote, efpecially if emaciation, which fucceeds to as great a degree in this difeafe as in the phthifis pulmonalis, has taken place.

In the milder fpecies of dyfentery, where gangrene does not fucceed to any confiderable extent of furface or depth, beyond the power of nature to caft it off in floughs without immediately affecting the life of the patient, and in thofe cafes where ulceration only fucceeds inflammation, there is a confiderable mitigation of the fymptoms, infomuch, that the difeafe may moft commonly be divided into the acute and chronic ftages.

In the firft, though the patient goes to ftool every five minutes, yet there is an obftinate conftipation, conſtipation *, the diſcharge is not excrementitious, or what has undergone digeſtion, but entirely conſiſts of blood and mucus, the effuſion and ſecretion of a part diſeaſed, cauſed by eroſion and anaſtomoſis of the blood veſſels, and increaſed glandular action. In the chronic ſtage it is a mixture of mucus, pus, and blood, with what has undergone digeſtion. The pain, at firſt, is chiefly cauſed by inflammation; afterwards it ariſes from ſcybala, or hardened fæces, paſſing through the ulcerated part, and therefore only recurs at longer intervals. The conſtipation ſeems to be owing to the ſtricture intercepting the periſtaltic motion; and though not ſo conſiderable in the chronic ſtage, continues more or leſs during the whole courſe of the diſeaſe.

In ſome inſtances ſlight hectic ſymptoms have ſupervened in the latter ſtage of the diſeaſe; and there is no doubt but a hectic, exquiſitely formed, as in the phthiſis pulmonalis, would

* "However, intolerable gripings, and a painful deſcent, as "it were, of all the bowels, always accompany the ſtools, "which are very frequent, and all mucous, not excrementi-"tious, unleſs that ſometimes an excrementitious one inter-"venes without any conſiderable pain."

*Swan's Tranſlation of Sydenham's Works, p.* 154.

take take place, if the matter generated and effufed had not fo free an exit by the anus. I fee no impropriety therefore in giving the name of *Tabes Dyfenterica* to the difeafe in the latter ftage.

*Treatment.*—We fhall divide it into that of the acute and that of the chronic ftage.—In the firft, an antiphlogiftic courfe is to be obferved. Emetics may fometimes be ufeful; and if the *Diathefis phlogiftica* prevail, venæfection will likewife be proper; but none of our patients were in a fituation to bear this evacuation.—Purgatives, and of thefe, falts and emetic tartar were found moft efficacious.

℞. Sal. cathartic. amar. ℥j.

Tartar emetic gr. ij.

Aquæ bullientis ℔j. M.

Cap. ℥ij. vel. ℥iij. 2$^{da}$ vel. 3$^{ia}$ quâque horâ, quotidie.

This method fhould be continued until the difeafe either fpontaneoufly ceafes by refolution, or the chronic ftage is known to have taken place by a great remiffion of fymptoms, and the appearance of white ftools five or fix times a day; but in debilitated and fcorbutic habits, purging muft be adapted to the ftrength of the patient. The purgative courfe anfwers

 almoft almoſt every purpoſe; the patient finds himſelf conſiderably relieved by a diſcharge of natural fæces; the ſtricture is leſſened, and inflammation abated. Rhubarb, ipecacuanha, and vitrum antimonii ceratum, were ſeverally ſubmitted to fair trials, but their adminiſtration was evidently attended with leſs ſucceſs than that of ſalts, &c.; and they appeared only to be uſeful inaſmuch as they conſtituted a part of the purgative courſe, and by no means as poſſeſſing a ſpecific virtue. The remainder of the treatment conſiſts in diluting plentifully with lemonade, barley water, white decoction, and the like, with ſtarch clyſters, and an opiate every night; for a good found ſleep not only frequently produces a remiſſion, but ſometimes a complete criſis. The opiate we chiefly uſed was a mixture of ſpir. mindereri, vin. antimon. and tinct. thebaic. which is perhaps the beſt at the beginning of the diſeaſe. Where the purgative courſe is ſtrictly followed, and we ſee by inſpection that the fæces are freely diſcharged, opiates are very ſafe and efficacious*. Bliſters were never tried.

Though

* After bleeding, Sydenham exhibited an opiate; as alſo after every purge, which he gave on alternate days: on the intermediate

Though it may appear inconfiftent to give cathartics fo freely to a patient already purging violently, yet it muft always be remembered that this is a deception; that the difcharge is only the fecretion or effufion of a part difeafed, and not excrementitious, or what has undergone digeftion; and that the latter, if fuffered to remain pent up above, will, by its increafing bulk, acrimony, and putrefaction, increafe every fymptom of the difeafe. It is neceffary, therefore, to infpect the ftools frequently; for, by what is difcharged, we can beft judge of what procefs is going on within.

In the chronic ftage, purging to fuch an extent is not neceffary, but evidently pernicious, by inducing too great debility. It is however neceffary to procure one or two natural ftools daily: the vis vitæ muft be fupported, and the ulcerations healed. To fulfil thefe indications, the following plan feemed to anfwer beft:—

intermediate days he gave the opiate twice. He fays, that after the third purge the cure depends entirely on giving an opiate twice or thrice a day; and moreover obferves, that in thofe conftitutions of the air which have a lefs tendency to promote this difeafe, omitting the evacuations above fpecified, it may be cured with laudanum only.

*Swan's Tranflation of Sydenham's Works, p.* 662 *and* 663.

A watery infufion of rhubarb * given every morning to procure the requifite excrementitious difcharge; for rhubarb, however improper in the firft ftage, is not fo in this.—An infufion † of bark, fl. chamæmeli, rad. gentian. and cort. aurant. or of bitters and aromatics, to ftrengthen the tone of the alimentary canal; P. Dovari ℈j. every night to procure reft; a decoction of ftarch and g. arabic, or milk, for common drink; a ftarch clyfter occafionally; and diet of fuch things as yield much nourifhment, and little fæces or fuch as may be of an irritating or putrefcent quality. A farinaceous and milk diet are to be ftrictly adhered to, with Madeira wine: every thing of an acefcent qua-

* Tincture of rhubarb and oil is recommended in old dyfenteries, attended with difcharges of blood.

*See Blane on the Difeafes of Seamen, p.* 449.

An equal quantity of conf. rofar. and fpermaceti, with half the quantity of an abforbent powder, is likewife recommended in old fluxes. *Ibid. p.* 450.

† In the lienteric ftate of the bowels, common in old fluxes, Dr. Blane thinks bark in every fhape hurtful, and recommends bitters, as fimarouba, quaffia, fl. chamæmeli, with aromatics, to obviate flatulence, which is a very common fymptom. He obferves likewife, that the compound officinals, as theriaca, diafcordium, pulv. e bolo c. &c., have been found of fervice. *Ibid. p.* 452.

lity,

ſity, as alſo animal food, muſt be avoided; for the acidity, ſpontaneouſly generated by the weakneſs of the digeſtive powers, frequently requires correcting by abſorbents; and the returning to a free uſe of animal food too ſoon, by overloading the ſtomach, has ſometimes brought on a relapſe. Aſtringents of every kind, and in almoſt every poſſible combination, were repeatedly tried, but with manifeſt diſadvantage. The recommendation of aſtringents with aromatic opiates, and particularly of torrefied rhubarb, ſo much depended on by Huxham, Hillary, and others, has evidently proceeded from a wrong theory. The adminiſtration of wax emulſions, balſ. Locatelli, and ſpermaceti, with gelatinous and mucilaginous drinks in the abraded ſtate of the inteſtines, are much more likely to be ſerviceable; as alſo the daily uſe of the clyſter*, ſo much extolled by Sydenham, to whoſe experience and candour great deference is due.

* Compoſed of half a pint of milk, and an ounce and a half of Venice treacle.

*See Swan's Tranſlation of Sydenham's Works, p.* 632.

